# Obstetrical Cases

**Published:** 1902-06

**Authors:** James H. Frinck

**Affiliations:** St. John, N. B.


### REPORTS OF CASES.

### OBSTETRICAL CASES.

By James H. Frinck, V.S.,
St. John, N. B.

I note in the reports of obstetrical cases published in the Journal from time to time that in cases of dystocia necessitating embryotomy more or less stress is laid upon the practice of carefully dissecting the skin from the limbs before attempting forcible removal. I think that the practitioner who follows this method puts a great deal of fatiguing work upon himself to very little purpose. Perhaps the most common form of malpresentation which is presented for treatment in the routine of practice is that by which the fore extremities are presented, with the head and neck toward the side. In the valuable breeding animals an attempt may be made, at retroversion, to deliver the fœtus alive. This is sometimes successful, but very often a great deal of valuable time is lost, whereby the mother and offspring are both lost. The value of the fœtus in the ordinary animal in such cases is, in my way of thinking, of very little value, and should receive scant consideration. For delivery: After placing the bedding, supports, etc., to your satisfaction, if the animal is in the recumbent position, tie its head securely to some fixture in the floor. Provide a small set of blocks or pulleys of metal—the smaller the better, if strong—one single and one double block, with a ring-bolt attachment to fasten on the sill or post immediately behind you; a pound or two of vaseline, preferable to oil in anointing the fœtus and canal; a large vessel containing a solution of bichloride of mercury, 1 to 500; knives, hooks, repellers, cords, sponges, and a few clean towels. Gently draw the leg toward you until the knee is clear of the vulva, remove at the knee-joint, adjust a noose on the lower end of the radius, secure to the pulleys. Have a retractor—made of metal, two and a half inches wide, absolutely smooth, spatula-shaped, round at the end, bent at right angles. This is passed in and along the wall of the vagina, above your working hand. The retractor is given to an assistant, who lifts away for clear work the relaxed walls of the vagina and the large volume of loose integument about the vulva; this is a great help. Slight strain being now put upon the pulleys, the elbow-joint comes into view and reach; disarticulate it. It is no trouble now to put your running noose on the lower end of the humerus. A slight strain on the pulleys, carry your knife to the shoulder, cut into the joint, rack the strain on the pulleys a little, and this section will come away easily. You now have a clear space in which to work. If you have difficulty now at getting the head of the fœtus, on account of being placed very far back, untie the patient's head and turn her completely over. A portion of the resistance being gone, the chances are that the head of the fœtus has dropped down, and it is ready at your hand to deliver. If not, remove the other foreleg the same as the first; then there is no difficulty. In securing the head with hooks avoid the lower jaw altogether; the slightest pull on it will smash it, and then if you have any further manipulation you are apt to get your hand or arm scratched with pieces of splintered bone or vicious little loose teeth. You can always get a good grip in the orbit, and the hook will hold if it is the right shape. Not the slightest danger need be anticipated from the cavity of the scapular articulations being exposed and doing internal mischief, as it will be found that they have retracted and are well hidden beneath muscular tissue at the point of the shoulder. I have time and again removed a fœtus in this manner in ten or fifteen minutes which had received hours of toil in the earlier days of my career by the subcutaneous methods laid down in text-books. It is almost a sin to send a young man out from college without any practical education in this line of practice. He often—far too often—becomes a burden to himself and not infrequently a source of amusement to the bucolic community into which fate has sent him.

